# Investigation of Association Between Hip Osteoarthritis Susceptibility Loci and Radiographic Proximal Femur Shape

**DOI:** 10.1002/art.39186

**Published:** 2015-07-28

**Authors:** Claudia Lindner, Shankar Thiagarajah, J. Mark Wilkinson, Kalliope Panoutsopoulou, Aaron G. Day‐Williams, The arcOGEN Consortium, Timothy F. Cootes, Gillian A. Wallis, John Loughlin, Nigel Arden, Fraser Birrell, Andrew Carr, Kay Chapman, Panos Deloukas, Michael Doherty, Andrew McCaskie, William E. R. Ollier, Ashok Rai, Stuart H. Ralston, Timothy D. Spector, Ana M. Valdes, Gillian A. Wallis, J. Mark Wilkinson, Eleftheria Zeggini

**Affiliations:** ^1^ University of ManchesterManchesterUK; ^2^ University of SheffieldSheffieldUK; ^3^ Wellcome Trust Sanger InstituteHinxtonCambridgeUK; ^4^ Wellcome Trust Sanger Institute, Hinxton, Cambridge, UK (current address: BiogenCambridgeMassachusetts)

## Abstract

**Objective:**

To test whether previously reported hip morphology or osteoarthritis (OA) susceptibility loci are associated with proximal femur shape as represented by statistical shape model (SSM) modes and as univariate or multivariate quantitative traits.

**Methods:**

We used pelvic radiographs and genotype data from 929 subjects with unilateral hip OA who had been recruited previously for the Arthritis Research UK Osteoarthritis Genetics Consortium genome‐wide association study. We built 3 SSMs capturing the shape variation of the OA‐unaffected proximal femur in the entire mixed‐sex cohort and for male/female‐stratified cohorts. We selected 41 candidate single‐nucleotide polymorphisms (SNPs) previously reported as being associated with hip morphology (for replication analysis) or OA (for discovery analysis) and for which genotype data were available. We performed 2 types of analysis for genotype–phenotype associations between these SNPs and the modes of the SSMs: 1) a univariate analysis using individual SSM modes and 2) a multivariate analysis using combinations of SSM modes.

**Results:**

The univariate analysis identified association between rs4836732 (within the ASTN2 gene) and mode 5 of the female SSM (*P* = 0.0016) and between rs6976 (within the GLT8D1 gene) and mode 7 of the mixed‐sex SSM (*P* = 0.0003). The multivariate analysis identified association between rs5009270 (near the IFRD1 gene) and a combination of modes 3, 4, and 9 of the mixed‐sex SSM (*P* = 0.0004). Evidence of associations remained significant following adjustment for multiple testing. All 3 SNPs had previously been associated with hip OA.

**Conclusion:**

These de novo findings suggest that rs4836732, rs6976, and rs5009270 may contribute to hip OA susceptibility by altering proximal femur shape.

Osteoarthritis (OA) is the most common form of human joint disease in the Western world 1. It is a major cause of pain and disability and has a huge socioeconomic impact, particularly in countries with an aging population 2. There are currently no effective predictive biomarkers for early OA and no available disease‐modifying therapies that alter its natural history. The development of methods for early diagnosis as well as new treatment options are therefore urgently needed in order to minimize the impact of the disease.

The progression of OA is associated with radiographic features such as degeneration of cartilage and consequent joint space narrowing. Recently, detailed morphometric analyses have been conducted to identify key radiographic features of bone shape that contribute to hip OA incidence and progression and that may serve as biomarkers for presymptomatic diagnosis and treatment evaluation 3, 4, 5. Those studies—based on radiographic features and measurements of bone geometry—indicate the importance of hip joint shape for susceptibility to hip OA. However, when studies focus primarily on individual morphometric measurements, the relationship between *global* hip joint shape and OA is not taken into account.

Statistical shape models (SSMs) provide a global representation of shape, which enables the quantitative description and analysis of bone shape variation across subjects 6. An SSM describes the shape of an object by the sum of a mean shape and a linear combination of a number of shape modes where the shape mode values vary between subjects. Gregory et al first described the application of SSMs for the analysis of proximal femur shape in their study of osteoporotic hip fractures 7. This approach has since been used in a number of studies to investigate the relationship between features of proximal femur shape and the onset, incidence, and progression of hip OA 8, 9, 10, 11. Such studies have also been extended to the analysis of potential genetic regulators of bone shape that may also contribute to hip OA susceptibility 12, 13. All of the above studies applied a univariate approach to analyzing the association between genetic loci and single SSM modes. However, a single SSM mode only describes one direction of shape variation in the population, whereas the overall shape consists of a subject‐specific combination of all shape model modes. Thus, approaches that capture shape more holistically may better represent the in vivo shape. We have therefore explored using a *combination of SSM modes* as a multivariate phenotype for genetic association studies, applying recently published methodology for genetic association analysis of multivariate/multiple phenotypes (e.g., for review, see refs. 14 and 15).

OA is a complex disease with environmental and genetic factors contributing to its susceptibility. The genetic component of primary hip OA has been estimated to be ∼60% 16. There is also evidence that OA is a highly polygenic disease with several genetic variants contributing to its susceptibility 17. Several studies have identified OA susceptibility loci that have been either confirmed at a genome‐wide significance level or that are suggestive and require replication (see Supplementary Table 1, available on the *Arthritis & Rheumatology* web site at http://onlinelibrary.wiley.com/doi/10.1002/art.39186/abstract). Since the shape of the proximal femur has previously been found to be associated with hip OA susceptibility, the assumption of this study was that a proportion of the genetic susceptibility to hip OA might be due to genetic variants that contribute to proximal femur shape. Loci that contribute to hip joint shape may, hence, also contribute to hip OA susceptibility and vice versa. However, limiting analyses to individual SSM modes may decrease the power to detect such loci.

In this study, we performed genotype–phenotype association analyses of SSM‐based proximal femur shape and candidate single‐nucleotide polymorphisms (SNPs) that had been associated previously with either hip morphology (with the aim of replicating previous suggestive association) or hip OA (with the aim of discovering, de novo, loci associated with hip joint shape that also contributed to hip OA susceptibility). This study included 1) a univariate association analysis between single SSM modes of proximal femur shape and candidate SNPs and 2) a multivariate association analysis between combined SSM modes of proximal femur shape and candidate SNPs.

## PATIENTS AND METHODS

### Samples

Anteroposterior (AP) pelvic radiographs were available from 2,224 subjects with hip OA recruited in stage 2 of the Arthritis Research UK Osteoarthritis Genetics (arcOGEN) Consortium genome‐wide association study (GWAS) 18. A list of the arcOGEN Consortium Investigators is provided in Appendix A.

**Figure 1:**
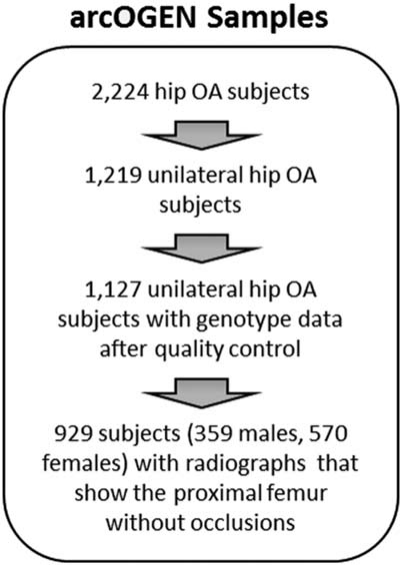
Overview of selection of study subjects from the Arthritis Research UK Osteoarthritis Genetics (arcOGEN) Consortium genome‐wide association study data set. OA = osteoarthritis.

Inclusion criteria and ethical approval for subject recruitment were as previously described 18. As shown in Figure 1, we selected radiographs of the OA‐unaffected hip joint (Kellgren/Lawrence score of <2 [19]) in patients with unilateral hip OA. The final data set comprised 929 unrelated subjects (570 women and 359 men) of Caucasian origin with AP pelvic radiographs on which the OA‐unaffected proximal femur was visible without any occlusions. Demographic data for each of the 929 subjects included body mass index (BMI) and age, the latter being defined as the age at first hip arthroplasty. BMI data were available for 546 women and 342 men, age data were available for 551 women and 346 men, and both BMI and age data were available for 528 women and 333 men.

### Genotyping, imputation, and quality control

Quality‐controlled genotype data were available for each of the 929 subjects from the arcOGEN Consortium GWAS.

A total of 813 subjects had been genotyped on Illumina Human610‐Quad BeadChips 18, and 116 subjects had been genotyped on Illumina HumanOmniExpress BeadChips 20. Quality control at the subject and SNP levels was performed as previously described 18, 20. For the 116 subjects genotyped on the HumanOmniExpress platform, imputed data were available. The imputation had been performed with IMPUTE2 version 2.1.2 on the European data from the 1000 Genomes Phase 1 interim release in December 2010 with files downloaded from the IMPUTE2 web site (http://mathgen.stats.ox.ac.uk/impute/data_download_1000G_2010_interim.html) 21. The imputation was run with the default IMPUTE2 parameters except for using a buffer size of 500 kb, an effective population size of 15,000, and the fix_strand_g option. Postimputation quality control was performed to ensure that all SNPs had an IMPUTE‐info score of >0.8 and a minor allele frequency (MAF) of >5%. We used GTOOL 22 to convert the imputed genotype probabilities into Plink 23 format by making hard genotype calls using a threshold of ≥0.9.

For the analyses in this study, we pooled the directly typed genotypes of the 813 subjects genotyped on the 610‐Quad platform and the imputed genotypes of the 116 subjects genotyped on the HumanOmniExpress platform. We refer to these below as the 2 arcOGEN genotype data sets.

### Candidate SNPs associated with hip OA or hip morphology

We systematically searched the literature to identify SNPs that had been associated with either hip OA or proximal femur morphology based on studies that were published up until December 2013. We included SNPs for which evidence of association had been confirmed at a genome‐wide level (*P* < 5.0 × 10^−8^) as well as SNPs showing a suggestive association as defined in the original study.

Supplementary Table 1 (available on the *Arthritis & Rheumatology* web site at http://onlinelibrary.wiley.com/doi/10.1002/art.39186/abstract) shows SNPs reported previously as being associated with either hip OA or proximal femur morphology. In total, we identified 42 SNPs as potential candidates for this study. Sixteen of the 42 SNPs were available in both arcOGEN genotype data sets after quality control. For the remaining candidate index SNPs, we identified 25 proxies (r^2^ or D′ > 0.85) that were present in both arcOGEN genotype data sets using the linkage disequilibrium (LD) search on http://www.broadinstitute.org/mpg/snap/ 24 based on the 1000 Genomes Pilot 1 data set for the CEU population (Utah residents with ancestry from northern and western Europe, from the collection of the Centre d'Étude du Polymorphisme Humain [http://www.cephb.fr/en/]). None of the original 42 candidate SNPs were in high LD with each other. In the genetic association analyses in this study, we included all candidate SNPs that were directly available for both arcOGEN genotype data sets as well as SNPs that were in high LD with one of the candidate SNPs and available for both data sets.

Supplementary Table 2 (available on the *Arthritis & Rheumatology* web site at http://onlinelibrary.wiley.com/doi/10.1002/art.39186/abstract) provides an overview of all candidate SNPs and summarizes the final 41 SNPs used in this study. For the 116 subjects for whom imputed data were available, 27 of the 41 SNPs were imputation basis SNPs, and all SNPs passed postimputation quality control.

### SSMs

Building an SSM is based on principal components analysis (PCA) and results in a number of shape model modes 6, with every mode defining a pattern of shape variation and all modes being orthogonal to each other. The first SSM mode accounts for the largest shape variation in the data set, the second mode for the largest amount of shape variation still remaining, and so on. For the purpose of analysis of bone shape variation, the inclusion of all SSM modes that together describe 95% of shape variation is accepted in the field as an appropriate cutoff to minimize noise 8, 10, 11.

For each radiograph, manual annotations were available outlining the contour of the OA‐unaffected proximal femur using 65 points as shown in Supplementary Figure 1 (available on the *Arthritis & Rheumatology* web site at http://onlinelibrary.wiley.com/doi/10.1002/art.39186/abstract). Based on these, we built 3 SSMs of the proximal femur: a mixed‐sex SSM (mean ± SD age 64.1 ± 15.0 years, mean ± SD BMI 27.4 ± 7.9 kg/m^2^), a female SSM (mean ± SD age 64.0 ± 15.0 years, mean ± SD BMI 27.7 ± 8.2 kg/m^2^), and a male SSM (mean ± SD age 64.3 ± 15.0 years, mean ± SD BMI 27.1 ± 7.3 kg/m^2^). Each of the SSMs provided a subject‐specific value for every shape model mode, describing the shape of the proximal femur based on the shape variation present in the given population. We built separate mixed‐sex and sex‐specific SSMs because of the difference in hip joint shape between men and women 25, 26 and so that selected candidate SNPs that had been identified previously in a sex‐stratified cohort could be tested in a population of that sex. For each SSM, 12 shape model modes explained 95% of the overall shape variation. Supplementary Table 3 (available on the *Arthritis & Rheumatology* web site at http://onlinelibrary.wiley.com/doi/10.1002/art.39186/abstract) summarizes the percentage of shape variation explained by each mode in every model. We used the Kolmogorov‐Smirnov test to verify that the data for each mode and every model were normally distributed.

### Univariate SNP association analysis

We performed a univariate SNP association analysis by testing each SNP in the population where the original association had been found (i.e., mixed‐sex, female, or male) and using the shape model mode values of the appropriate SSM. Hence, we tested each SNP for only 1 of the 3 sex subsets as specified in Supplementary Table 2 (available on the *Arthritis & Rheumatology* web site at http://onlinelibrary.wiley.com/doi/10.1002/art.39186/abstract). All associations between the shape modes and genotypes were estimated using linear regression (Plink command –linear) in an additive genetic model in Plink version 1.07 23.

To determine empirically the significance of the linear regression results, we performed 100,000 max(T) permutation tests independently for each SNP, randomly reassigning the phenotypes to each individual (Plink command –mperm). This resulted in 2 empirical estimates of significance for every test, a pointwise *P* value of the individual SNP (Plink variable EMP1) and a familywise *P* value that was corrected for all comparisons (Plink variable EMP2). The latter is a more stringent criterion for significance, and we considered SNPs with a familywise *P* value of less than 0.05 to show a statistically significant association. We also adjusted significance levels using the traditional Bonferroni correction when accounting for all SNPs and SSM modes tested. We used Quanto version 1.2.4 27 to calculate power estimates for our univariate analyses following an additive genetic model and assuming a Type I error rate of 5%.

### Multivariate SNP association analysis

We considered all shape model modes of an SSM to represent a multivariate phenotype and performed multivariate tests of association to identify SNPs that contribute to the shape of the proximal femur. We tested all SNPs for their association with a *combination* of all shape model modes of the appropriate SSM (i.e., mixed‐sex, female, or male) using the multivariate test of association implemented in Plink version 1.06p 28 (available from http://genepi.qimr.edu.au/staff/manuelF/multivariate/main.html). This implementation is based on canonical correlation analysis and tests for linear combinations of all phenotypes (in our case SSM modes) that are most associated with the genotype at the SNP. When performing permutation tests using the multivariate test of association implemented in Plink, only pointwise, but not familywise, *P* values are reported. Therefore, we estimated the significance level of our multivariate results using traditional Bonferroni correction, which accounted for all 41 SNPs tested. We considered SNPs with a *P* value of less than 0.001 to show a statistically significant association. All plots and calculations were made using custom code developed in C++ and MatLab R2012a.

## RESULTS

### SNP association analysis using univariate shape data

**Figure 2:**
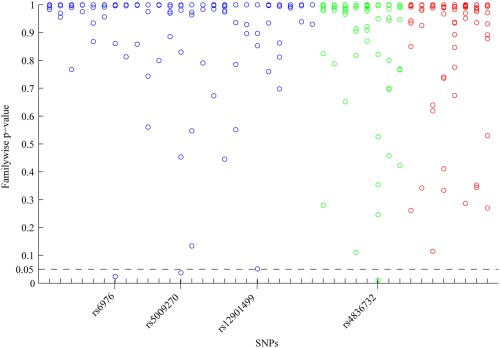
Familywise *P* values for all 492 tests (41 single‐nucleotide polymorphisms [SNPs], 12 statistical shape model [SSM] modes) for association between univariate SSM modes of the proximal femur and candidate SNPs. All familywise *P* values are before adjustment for covariates and were obtained by running 100,000 permutations. The expected level for significance was a familywise *P* value of less than 0.05, which is indicated by the dashed line. Three SNPs (rs6976, rs5009270, and rs4836732) reached this significance level and 1 SNP (rs12901499) was just above it. Every SNP was tested following an additive genetic model in the stratum where the original association had been found (i.e., mixed‐sex [blue], female [green], or male [red]).

Of the 41 SNPs, 25 were tested against the mixed‐sex SSM modes, 8 against the female SSM modes, and 8 against the male SSM modes (see Supplementary Table 2, available on the *Arthritis & Rheumatology* web site at http://onlinelibrary.wiley.com/doi/10.1002/art.39186/abstract). The familywise *P* values are diagrammatically represented in Figure 2 for all SNPs before adjustment for age and BMI. The figure shows that before adjustment for covariates, 3 SNPs (rs6976, rs4836732, and rs5009270) had a familywise *P* value of less than 0.05 and 1 SNP (rs12901499) had a familywise *P* value of less than 0.052 when tested using 100,000 permutations following an additive genetic model. After adjustment for age and BMI, only rs4836732 remained significant at a familywise significance level of less than 0.05 (see Table 1). However, following Bonferroni correction for all 492 tests (41 SNPs, 12 SSM modes), none of the identified signals reached the expected pointwise significance level of less than 0.0001 before or after adjustment for age and BMI.

**Table 1:** Association between univariate SSM modes of the proximal femur and SNPs previously reported to be associated with hip osteoarthritis or hip morphology^a^

SNP/chromosome		Pointwise *P*	Familywise *P*	Sex subset	SSM mode	Minor allele	MAF
Before adjustment for covariates							
rs6976/3		0.00096	0.02355	Mixed	7	T	0.40
rs6976/3^b^		0.00028	0.00455	Mixed	7	T	0.40
rs5009270/7		0.0015	0.03742	Mixed	9	A	0.33
rs4836732/9		0.00159	0.01096	Female	5	T	0.48
After adjustment for age and BMI							
rs6976/3		0.00208	0.05025	Mixed	7	T	0.40
rs6976/3^b^		0.00091	0.02064	Mixed	7	T	0.40
rs5009270/7		0.00608	0.1278	Mixed	9	A	0.33
rs4836732/9		0.00493	0.03368	Female	5	T	0.48

a The expected level for significance was a familywise *P* value of less than 0.05. All results were obtained running 100,000 permutation tests following an additive genetic model (if not stated otherwise). SSM = statistical shape model; SNPs = single‐nucleotide polymorphisms; MAF = minor allele frequency; BMI = body mass index.

b Results were obtained following a dominant, rather than an additive, genetic model.

Analysis of the mean SSM mode values per genotype for the above SNPs revealed that the effect caused by rs6976 on the values of SSM mode 7 might not be additive (mean mode value for T/T, −0.0012; for T/C, −0.0011; and for C/C, 0.0020). Therefore, we tested rs6976 for association with SSM mode 7 in the mixed‐sex model assuming dominance for the minor allele (Plink command –dominant). Following a dominant model, rs6976 was significantly associated with SSM mode 7 before and after adjustment for age and BMI (see Table 1).

**Figure 3:**
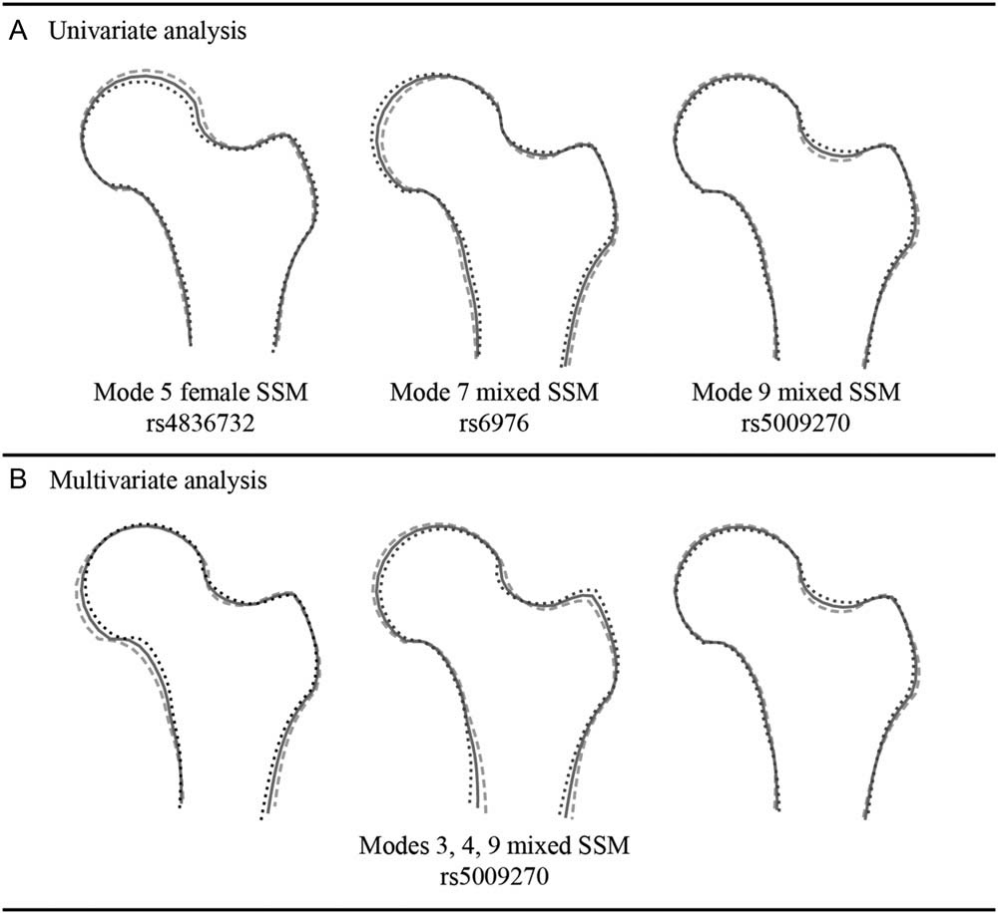
Statistical shape model (SSM) modes associated with candidate single‐nucleotide polymorphisms in this study from the univariate analysis (**A**) and the multivariate analysis (**B**). “Female” and “mixed” refer to the SSM used in the association analysis. Each drawing shows the mean (solid line) ± 2.5 SD (dotted and dashed lines).

Two of the 3 SNPs in Table 1 were previously reported as being associated with hip OA–related joint replacement (rs6976 and rs4836732), and the other SNP was previously reported as being associated with hip OA (rs5009270). Figure 3A shows the SSM modes for these 3 SNPs. The association of rs4836732 with mode 5 in the female model indicates that this SNP might have an effect on the superior orientation and size of the femoral head. A similar picture is given by the association of rs6976 with mode 7 in the mixed‐sex model, although this seems to represent overall femoral head size variation.

### SNP association analysis using multivariate shape data

Each SNP was tested for association with all 12 modes of the appropriate SSM (i.e., mixed‐sex, female, or male) simultaneously and following an additive genetic model. None of the 41 SNPs reached the expected significance level of less than 0.001 before or after adjustment for age and BMI. The 5 SNPs with the lowest *P* values (before/after adjustment for covariates) were rs5009270 (0.02/0.03), rs4836732 (0.02/0.07), rs6976 (0.06/0.13), rs1516893 (0.09/0.10), and rs12901499 (0.11/0.15). All the remaining SNPs showed *P* values of greater than 0.2 before adjustment for covariates and of greater than 0.1 after adjustment for covariates.

We investigated the contribution of the individual SSM modes to the overall association for each of the above 5 SNPs. Table 2 gives a summary of the individual contributions. For each of the 5 SNPs, we then repeated the multivariate analysis including only the 3 SSM modes contributing the most (Table 2), as noncontributing modes might decrease rather than increase the power of the multivariate approach 29. We chose the 3 SSM modes contributing the most as in all cases their contribution values were different from the remaining loading values across all SNPs. The results are shown in Table 3. Both rs5009270 and rs4836732 had *P* values of less than 0.001 before adjustment for covariates, and rs5009270 also reached this significance level after adjustment for age and BMI. All multivariate tests were performed following an additive genetic model, as other genetic models are not currently included in the multivariate test of association in Plink. Therefore, the multivariate results for rs6976 might be undervalued since the univariate analysis indicated a dominant effect of this SNP on SSM mode 7.

**Table 2:** Absolute loading values of individual SSM modes for the 5 SNPs that showed the most significant association in the multivariate analysis, indicating the contribution of each SSM mode to this association

	SSM mode											
SNP	1	2	3	4	5	6	7	8	9	10	11	12
rs5009270	0.12	0.22	0.37^a^	0.46^a^	0.01	0.03	0.01	0.29	0.64^a^	0.26	0.14	0.02
rs4836732	0.06	0.04	0.01	0.34	0.66^a^	0.02	0.04	0.39^a^	0.12	0.26	0.20	0.42^a^
rs6976	0.16	0.12	0.16	0.12	0.39^a^	0.26	0.73^a^	0.12	0.25	0.28^a^	0.08	0.07
rs1516893	0.20	0.09	0.34	0.09	0.16	0.16	0.63^a^	0.13	0.02	0.35^a^	0.49^a^	0.02
rs12901499	0.08	0.14	0.25	0.16	0.10	0.72^a^	0.40^a^	0.13	0.41^a^	0.07	0.02	0.18

a Indicates the 3 most contributing statistical shape model (SSM) modes for each single‐nucleotide polymorphism (SNP).

Figure 3B shows the 3 SSM modes that contribute to the significant association with rs5009270. This association with SSM modes 3, 4, and 9 in the mixed‐sex model indicates that rs5009270 might have an effect on femoral neck width. Supplementary Figure 2 (available on the *Arthritis & Rheumatology* web site at http://onlinelibrary.wiley.com/doi/10.1002/art.39186/abstract) shows the 3 SSM modes that contribute to the association with each of the remaining 4 SNPs.

## DISCUSSION

We have identified 3 SNPs that are significantly associated with proximal femur morphology in a cohort of subjects with hip OA, before and after adjustment for age and BMI. The SNP rs4836732 (within the ASTN2 gene) was found to be associated with SSM mode 5 in a female model following a univariate additive genetic association analysis, the SNP rs6976 (within the GLT8D1 gene) was found to be associated with SSM mode 7 in a mixed‐sex model following a univariate dominant genetic association analysis, and the SNP rs5009270 (near the IFRD1 gene) was found to be associated with the combination of SSM modes 3, 4, and 9 in a mixed‐sex model following a multivariate additive genetic association analysis. We also repeated the univariate and multivariate analyses adjusting for height in view of prior reports of an excess of shared signals between height and OA 30. We found, however, that none of the associations reported in Tables 1 and 3 were dependent on height (see Supplementary Tables 4 and 5, available on the *Arthritis & Rheumatology* web site at http://onlinelibrary.wiley.com/doi/10.1002/art.39186/abstract). Given that all 3 SNPs had previously been reported to be associated with hip OA 18, 20 and also showed significant association with proximal femur shape in the present study (a completely different study design of SSM‐based quantitative trait analysis), we hypothesize that these SNPs contribute to hip OA susceptibility by altering proximal femur morphology.

**Table 3:** Multivariate association between the 3 most contributing SSM modes of the proximal femur and SNPs previously reported to be associated with hip osteoarthritis or hip morphology^a^

SNP/chromosome		*P*	Sex subset	SSM modes	Minor allele	MAF
Before adjustment for covariates						
rs6976/3		0.00149	Mixed	5, 7, 10	T	0.40
rs5009270/7		0.00039	Mixed	3, 4, 9	A	0.33
rs4836732/9		0.00044	Female	5, 8, 12	T	0.48
rs1516893/9		0.00237	Mixed	7, 10, 11	A	0.12
rs12901499/15		0.00158	Mixed	6, 7, 9	A	0.44
After adjustment for age and BMI						
rs6976/3		0.00555	Mixed	5, 7, 10	T	0.40
rs5009270/7		0.00040	Mixed	3, 4, 9	A	0.33
rs4836732/9		0.00347	Female	5, 8, 12	T	0.48
rs1516893/9		0.00363	Mixed	7, 10, 11	A	0.12
rs12901499/15		0.00219	Mixed	6, 7, 9	A	0.44

a The expected level for significance after Bonferroni correction was a *P* value of less than 0.001. All results were obtained following an additive genetic model. SSM = statistical shape model; SNP = single‐nucleotide polymorphism; MAF = minor allele frequency; BMI = body mass index.

Investigating the shape variation explained by the associated SSM modes suggests that rs5009270 contributes to femoral neck width and that rs4836732 and rs6976 contribute to femoral head size. The first finding is consistent with previous findings which show that a wider femoral neck is associated with hip OA 3. Furthermore, both rs4836732 and rs6976 were found to be associated with hip OA in subjects who had undergone total joint replacement 18. With the increasing evidence that total hip replacement can be predicted based on radiographic proximal femur shape (independently of radiographic hip OA at baseline) 5, 10, 11, this supports our hypothesis that these SNPs contribute to hip OA susceptibility by affecting proximal femur shape. The proposed functions of the genes nearest to the associated SNPs were reviewed when first identified (see Supplementary Table 1, available on the *Arthritis & Rheumatology* web site at http://onlinelibrary.wiley.com/doi/10.1002/art.39186/abstract), and no further information has been published in support of these genes as obvious candidates for proximal femur morphology or hip OA. Further, it has not been confirmed which genes contribute to susceptibility as, for example, rs6976 is in perfect LD with rs11177 (within the GNL3 gene) in a gene‐dense LD block (>30 genes) 18. Therefore, it is not known at this stage what the molecular basis might be for the association with proximal femur shape.

Our rationale for applying a phenotype‐based *multivariate* approach to genetic association analysis was that, if there is correlation between several components of a multivariate phenotype and these contribute to a signal, then the statistical power of the analysis may increase 15 and lead to a reduction in multiple testing 29. By the definition of PCA, all modes of an SSM are linearly independent. However, this study was based on the assumption that a subset of all modes of a model might be correlated with respect to a specific genotype. Therefore, to test this hypothesis, we performed multivariate SNP association analyses by testing for association between a genotype and a combination of SSM modes.

The identification of rs5009270 as associated with a combination of SSM modes suggests that the effect of proximal femur shape susceptibility SNPs may not be limited to a single aspect of shape. Using combinations of SSM modes in the analysis of radiographic bone shape is a promising approach to uncover new associations with disease and the underlying cause. All SNPs that were suggestive in our univariate SNP association analysis were also suggestive in our multivariate SNP association analysis—where the SSM mode of association identified in the univariate analysis was also the mode contributing the most to the association in the multivariate analysis (see loading values in Table 2). This confirms that the multivariate analysis can be used to identify the main SSM mode of association requiring just a single test per SNP rather than testing each SNP for each SSM mode individually. Once association is identified by the multivariate analysis, a further single univariate analysis for the mode contributing the most can be performed to test for the significance of the association. Hence, the multivariate analysis reduces the number of tests performed and thereby increases the power of the analysis. In addition, the multivariate analysis may identify associations that are not clearly attributable to a single SSM mode but rather to a combination of SSM modes—which would not be identified by testing for individual SSM modes only as in the univariate approach. Therefore, in future studies of the underlying genetics of bone shape, we suggest first using a multivariate approach to SNP association analysis to identify potential SNPs and SSM modes of association, followed by a univariate approach to investigate and confirm the findings.

A limitation of this study is that it relies on AP pelvic radiographs, which only give a 2‐dimensional projection of the 3‐dimensional proximal femur. The projected radiographic shape of the proximal femur may vary due to subject positioning during image acquisition (e.g., due to pelvic or leg rotation). Therefore, no definite conclusions can be drawn as to whether the observed genotype–phenotype associations relate to the true physical shape and/or the projected radiographic shape, the latter of which might be altered by disease (e.g., due to pain or restricted movement). However, previous studies have shown radiographic proximal femur shape to be a predictor of the onset of hip OA before the presence of any clinical or radiographic signs of disease 3, 5, 8. In addition, the present study was conducted on proximal femurs that were free of radiographic signs of hip OA. Thus, we hypothesize that the identified susceptibility loci have an effect on proximal femur morphogenesis, resulting in altered proximal femur shape that predisposes to hip OA.

Previously reported SSM‐based genotype–phenotype associations of hip joint shape with SNPs in the DIO2 and FRZB regions 12, 13 were not replicated in this study. The reasons for this might be that the SSMs capture different aspects of hip joint shape, that the significance of association in the original study had not been corrected for multiple testing (for DIO2 variants [12]), or that the current study was underpowered due to sample size (for FRZB variants [13]). Since power to detect any of the examined loci depends largely on their anticipated effect size (and other parameters), which were unknown (DIO2) or not standardized (FRZB), we instead conducted theoretical power calculations for the variants examined in our study. In these calculations, we used as a guide the effect sizes of our reported associations with modes 5, 7, and 9. Our power calculations showed that for these effect sizes, our univariate analyses had >80% power to detect an association for SNPs with a MAF of ≥0.25 at an alpha level of less than 0.05 across all strata examined (see Supplementary Table 6, available on the *Arthritis & Rheumatology* web site at http://onlinelibrary.wiley.com/doi/10.1002/art.39186/abstract). We expect modest increases in power for higher SSM mode numbers since, according to the definition of PCA, the standard deviation of the modes decreases with increasing mode numbers. For example, all SNPs with power x to detect an association with SSM mode 5 (assuming an effect size of −0.0029) will have power > x to detect an association with SSM modes 6–12. It is noteworthy that the examined effect sizes are representative of the typical small‐to‐modest effect sizes that have been observed for all robustly established hip OA loci to date.

This study was conducted on radiographs of the OA–unaffected hip joint of subjects with unilateral hip OA on the assumption that the OA–unaffected side is a good predictor of the shape of the affected side before disease. This assumption was based on our prior analyses of the 2‐dimensional radiographic shape of left and right proximal femurs as captured by an SSM 31. Assuming radiographic proximal femur symmetry 31 before the onset of disease, this raises questions as to what determines which side (left/right) will first develop OA, whether carriers of the risk allele of a susceptibility SNP are more prone to develop bilateral hip OA over time, and at what stage the morphology of the femur reflects changes as a consequence of disease rather than a predisposition. Future longitudinal studies will be needed to explore the interplay between genetically determined proximal femur morphology and the onset/progression of disease.

We have identified several significant and suggestive associations between hip OA susceptibility SNPs and radiographic proximal femur shape as captured by SSMs. Further replication would be advantageous to boost power and provide validation of these associations additional between robustly established hip OA loci and proximal femur shape. In future studies of bone shape, we suggest the use of a combination of multivariate and univariate SNP association analyses both to increase power and to enable the identification of associations with a combination of shape model modes. These methods and approaches can be applied as further associations are reported for OA (for example, as reported by Evans et al [32]). Functional studies will also be needed to determine the causative nature of the identified associations.

## AUTHOR CONTRIBUTIONS

All authors were involved in drafting the article or revising it critically for important intellectual content, and all authors approved the final version to be published. Dr. Lindner had full access to all of the data in the study and takes responsibility for the integrity of the data and the accuracy of the data analysis.

### Study conception and design

Lindner, Thiagarajah, Cootes, Wallis.

### Acquisition of data

Lindner, Thiagarajah, Wilkinson, Day‐Williams.

### Analysis and interpretation of data

Lindner, Wilkinson, Panoutsopoulou, Cootes, Wallis.

## ADDITIONAL DISCLOSURES

Author Day‐Williams is an employee of Biogen.

## Supporting information

Supplementary Figure 1: Annotation example outlining the proximal femur using 65 points.
Supplementary Figure 2: Shape model modes of multivariate results. “Mixed/female/male SSM” refers to the SSM used in the association analysis. Each figure shows the average (–) and ±2.5 standard deviations.
Supplementary Table 1: Summary of SNPs associated with hip OA or proximal femur morphology (TJR = total joint replacement (hip/knee); THR = total hip replacement; OA = osteoarthritis; TKR = total knee replacement; JSW = joint space width; SSM = statistical shape model; FNAL = femoral neck axis length; FNW = femoral neck width; FSW = femoral shaft width; BR = buckling ratio; FNSA = femoral neck shaft angle; NNSM = narrow neck section modulus). “Expected significance level” is the p‐value threshold used in the original study for the results to be significant.
Supplementary Table 2: Candidate arcOGEN SNPs selected for this study: Original SNPs are those reported in the literature as associated with hip osteoarthritis or proximal femur morphology. “arcOGEN SNP in LD” lists the final 41 SNPs included in this study which includes those in high LD (defined as r^2^ or D' >0.85) with the “Original SNP”. “Gender‐subset” specifies the gender strata where association was found in the original study and hence the gender stratum used in this study.
Supplementary Table 3: Percentage of shape variation explained by the modes of the mixed‐gender, female and male SSMs (n = number of subjects included in the model).
Supplementary Table 4: Association between univariate SSM modes of the proximal femur and previously reported hip osteoarthritis or morphology susceptibility SNPs (MAF = minor allele frequency) after adjustment for height as well as for age, BMI and height. The expected level for significance was a familywise p‐value <0.05. All results were obtained running 100,000 permutation tests following an additive genetic model (if not stated otherwise).
Supplementary Table 5: Multivariate association between the three most contributing SSM modes of the proximal femur and previously reported hip osteoarthritis or morphology susceptibility SNPs (MAF = minor allele frequency) after adjustment for height as well as for age, BMI and height. The expected level for significance after Bonferroni correction was a p‐value <0.001. All results were obtained following an additive genetic model.
Supplementary Table 6: Summary of power calculations for detecting an association with SSM modes 5 (effect size of rs4836732: −0.002899), 7 (effect size of rs6976: −0.001837) or 9 (effect size of rs5009270: −0.001611), assuming a Type I error rate of 5%. All effect sizes were taken from the univariate analyses following an additive genetic model (MAF = minor allele frequency).

## APPENDIX A THE arcOGEN CONSORTIUM INVESTIGATORS

The arcOGEN Consortium Investigators are as follows: John Loughlin, Principal Investigator, Newcastle University, Newcastle, UK; Nigel Arden, University of Oxford, Oxford, UK, and University of Southampton, Southampton, UK; Fraser Birrell, Newcastle University, Newcastle, UK; Andrew Carr, University of Oxford, Oxford, UK; Kay Chapman, University of Oxford, Oxford, UK; Panos Deloukas, Wellcome Trust Sanger Institute, Hinxton, Cambridge, UK; Michael Doherty, University of Nottingham, Nottingham, UK; Andrew McCaskie, Newcastle University, Newcastle, UK; William E. R. Ollier, University of Manchester, Manchester, UK; Ashok Rai, Worcestershire Acute Hospitals NHS Trust, Worcester, UK; Stuart H. Ralston, University of Edinburgh, Edinburgh, UK; Timothy D. Spector, King's College London, London, UK; Ana M. Valdes, King's College London, London, UK; Gillian A. Wallis, University of Manchester, Manchester, UK; J. Mark Wilkinson, University of Sheffield, Sheffield, UK; and Eleftheria Zeggini, Wellcome Trust Sanger Institute, Hinxton, Cambridge, UK.
