# Supplementary material for: Investigation of Association Between Hip Osteoarthritis Susceptibility Loci and Radiographic Proximal Femur Shape
Source: Arthritis Rheumatol. 2015 Jul 28;67(8):2076–84. doi: 10.1002/art.39186 (PMC4864451; doi:10.1002/art.39186)
Supplement: Supplementary file 1 — Supplementary Figure 1: Annotation example outlining the proximal femur using 65 points. Supplementary Figure 2: Shape model modes of multivariate results. “Mixed/female/male SSM” refers to the SSM used in the association analysis. Each figure shows the average (–) and ±2.5 standard deviations. Supplementary Table 1: Summary of SNPs associated with hip OA or proximal femur morphology (TJR = total joint replacement (hip/knee); THR = total hip replacement; OA = osteoarthritis; TKR = total knee replacement; JSW = joint space width; SSM = statistical shape model; FNAL = femoral neck axis length; FNW = femoral neck width; FSW = femoral shaft width; BR = buckling ratio; FNSA = femoral neck shaft angle; NNSM = narrow neck section modulus). “Expected significance level” is the p‐value threshold used in the original study for the results to be significant. Supplementary Table 2: Candidate arcOGEN SNPs selected for this study: Original SNPs are those reported in the literature as associated with hip osteoarthritis or proximal femur morphology. “arcOGEN SNP in LD” lists the final 41 SNPs included in this study which includes those in high LD (defined as r2 or D' >0.85) with the “Original SNP”. “Gender‐subset” specifies the gender strata where association was found in the original study and hence the gender stratum used in this study. Supplementary Table 3: Percentage of shape variation explained by the modes of the mixed‐gender, female and male SSMs (n = number of subjects included in the model). Supplementary Table 4: Association between univariate SSM modes of the proximal femur and previously reported hip osteoarthritis or morphology susceptibility SNPs (MAF = minor allele frequency) after adjustment for height as well as for age, BMI and height. The expected level for significance was a familywise p‐value <0.05. All results were obtained running 100,000 permutation tests following an additive genetic model (if not stated otherwise). Supplementary Table 5: Multivariate asso [file ART-67-2076-s001.docx]

**Investigation of Association between Hip Osteoarthritis Susceptibility Loci and Radiographic Proximal Femur Shape**

**Supplementary Material**

Contents

1. Supplementary figures
2. Supplementary tables

**1. Supplementary figures**


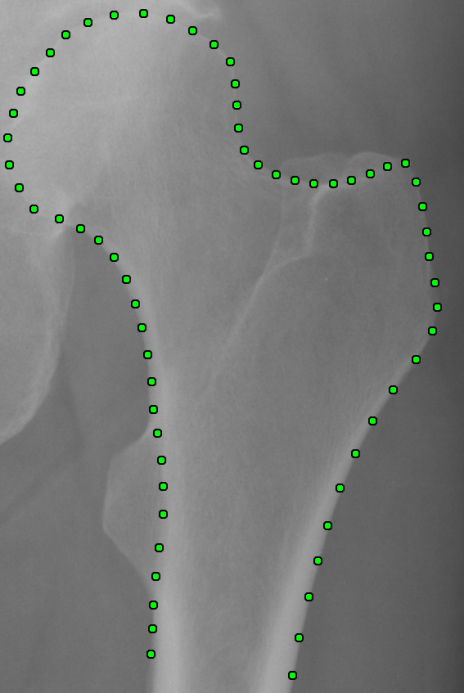


Figure S1: Annotation example outlining the proximal femur using 65 points.


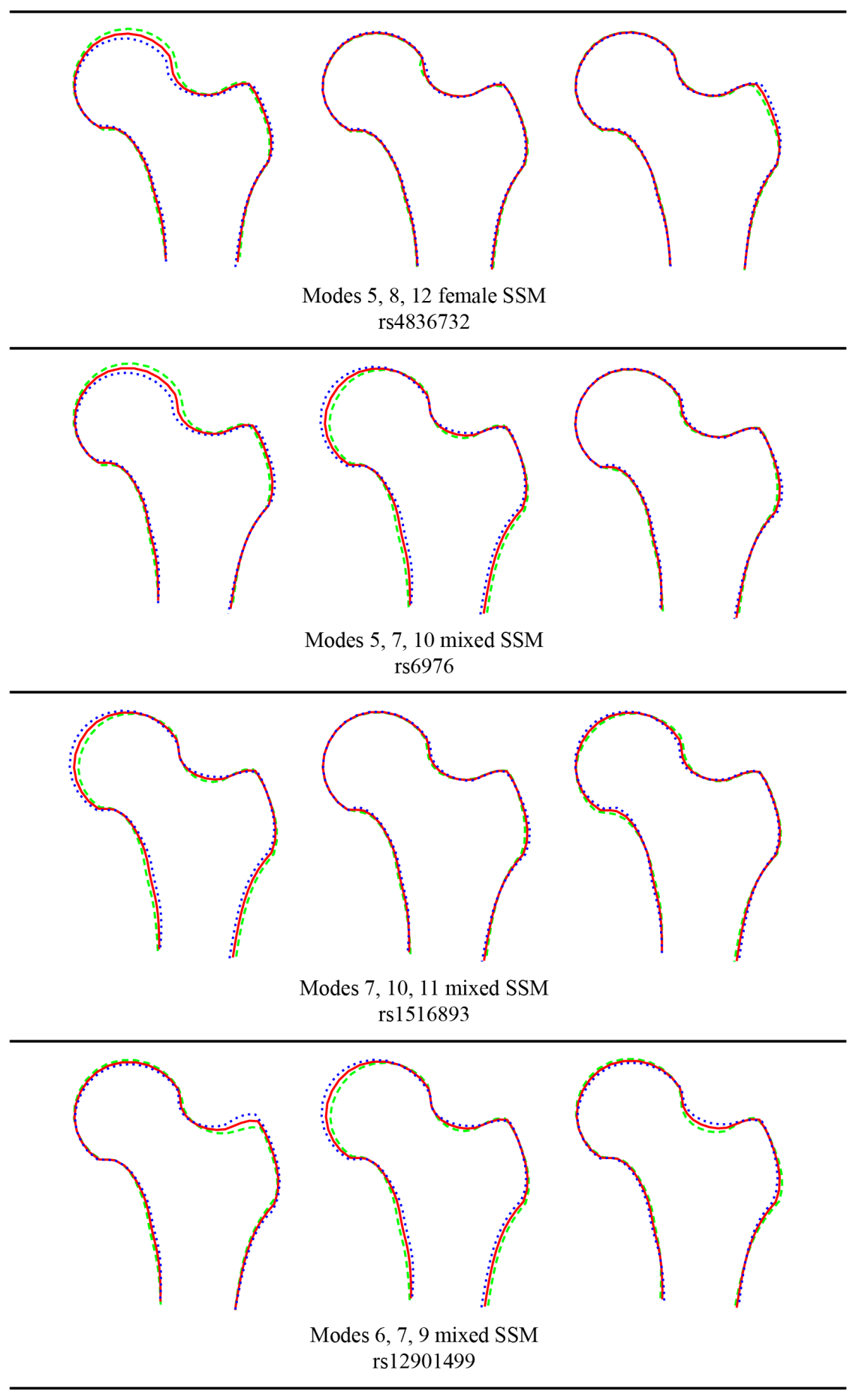


Figure S2: Shape model modes of multivariate results. “Mixed/female/male SSM” refers to the SSM used in the association analysis. Each figure shows the average (–) and ±2.5 standard deviations.

**2. Supplementary tables**

Table S1: Summary of SNPs associated with hip OA or proximal femur morphology (TJR = total joint replacement (hip/knee); THR = total hip replacement; OA = osteoarthritis; TKR = total knee replacement; JSW = joint space width; SSM = statistical shape model; FNAL = femoral neck axis length; FNW = femoral neck width; FSW = femoral shaft width; BR = buckling ratio; FNSA = femoral neck shaft angle; NNSM = narrow neck section modulus). “Expected significance level” is the p-value threshold used in the original study for the results to be significant.

| SNP | Reference | Nearest gene(s) | | Location | | Stratum | | Expected | | Observed | |
| --- | --- | --- | --- | --- | --- | --- | --- | --- | --- | --- | --- |
|  |  |  | |  | |  | | significance level | | p-value | |
| rs6976 | arcOGEN Consortium and arcOGEN | | GLT8D1 | | 3p21 | | TJR, mixed-gender | | < 5.0 × 10^−08^ | | 7.24 × 10^−11^ |
|  | Collaborators [1] | |  | |  | |  | |  | |  |
| rs4836732 | arcOGEN Consortium and arcOGEN | | ASTN2 | | 9q33 | | THR, female | | < 5.0 × 10^−08^ | | 6.11 × 10^−10^ |
|  | Collaborators [1] | |  | |  | |  | |  | |  |
| rs9350591 | arcOGEN Consortium and arcOGEN | | FILIP1, SENP6 | | 6q14 | | Hip OA, mixed-gender | | < 5.0 × 10^−08^ | | 2.42 × 10^−09^ |
|  | Collaborators [1] | |  | |  | |  | |  | |  |
| rs10492367 | arcOGEN Consortium and arcOGEN | | KLHDC5, | | 12p11 | | Hip OA, mixed-gender | | < 5.0 × 10^−08^ | | 1.48 × 10^−08^ |
|  | Collaborators [1] | | PTHLH | |  | |  | |  | |  |
| rs835487 | arcOGEN Consortium and arcOGEN | | CHST11 | | 12q23 | | THR, mixed-gender | | < 5.0 × 10^−08^ | | 1.64 × 10^−08^ |
|  | Collaborators [1] | |  | |  | |  | |  | |  |
| rs12107036 | arcOGEN Consortium and arcOGEN | | TP63 | | 3q26 | | TKR, female | | < 5.0 × 10^−08^ | | 6.71 × 10^−08^ |
|  | Collaborators [1] | |  | |  | |  | |  | |  |
| rs8044769 | arcOGEN Consortium and arcOGEN | | FTO | | 16q12 | | Hip/knee OA, female | | < 5.0 × 10^−08^ | | 6.85 × 10^−08^ |
|  | Collaborators [1] | |  | |  | |  | |  | |  |
| rs10948172 | arcOGEN Consortium and arcOGEN | | SUPT3H, | | 6p21 | | Hip/knee OA, male | | < 5.0 × 10^−08^ | | 7.92 × 10^−08^ |
|  | Collaborators [1] | | CDC5L | |  | |  | |  | |  |
| rs12982744 | Evangelou et al. [2] | | DOT1L | | 19p13 | | Hip JSW, male | | < 5.0 × 10^−08^ | | 7.8 × 10^−09^ |
| rs6094710 | Evangelou et al. [3] | | NCOA3 | | 20q13 | | Hip OA, mixed-gender | | < 5.0 × 10^−08^ | | 7.9 × 10^−09^ |
| rs5009270 | Evangelou et al. [3] | | IFRD1 | | 7q31 | | Hip OA, mixed-gender | | < 5.0 × 10^−08^ | | 9.0 × 10^−07^ |
| rs3757837 | Evangelou et al. [3] | | CAMK2B | | 7p13 | | Hip OA, male | | < 5.0 × 10^−08^ | | 2.2 × 10^−06^ |
| rs288326 | Baker-Lepain et al. [4] | | FRZB | | 2q32 | | SSM proximal femur, female | | < 0.05 | | 0.019 |
| rs7775 | Baker-Lepain et al. [4] | | FRZB | | 2q32 | | SSM proximal femur, female | | < 0.05 | | 0.019 |
| rs1974201 | Cheung et al. [5] | | ENPP1 | | 6q23 | | FNAL, FNW, FSW, mixed-gender | | < 8.1 × 10^−05^ | | 2.3 × 10^−04^ |
| rs4931462 | Castano-Betancourt et al. [6] | | OVOS2 | | 12p11 | | Hip JSW, mixed-gender | | < 5.0 × 10^−08^ | | 4.3 × 10^−07^ |
| rs10948155 | Castano-Betancourt et al. [6] | | SUPT3H | | 6p21 | | Hip JSW, mixed-gender | | < 5.0 × 10^−08^ | | 7.7 × 10^−07^ |
| rs11665774 | Castano-Betancourt et al. [6] | | SLC27A1 | | 19p13 | | Hip JSW, mixed-gender | | < 5.0 × 10^−08^ | | 4.0 × 10^−06^ |
| rs11738020 | Castano-Betancourt et al. [6] | | PIK3R1 | | 5q13 | | Hip JSW, mixed-gender | | < 5.0 × 10^−08^ | | 5.5 × 10^−06^ |
| rs2380165 | Castano-Betancourt et al. [6] | | BLM | | 15q26 | | Hip JSW, mixed-gender | | < 5.0 × 10^−08^ | | 5.6 × 10^−06^ |
| rs11206937 | Castano-Betancourt et al. [6] | | TRIT1, BMP8B | | 1p34 | | Hip JSW, mixed-gender | | < 5.0 × 10^−08^ | | 7.3 × 10^−06^ |
| rs12907468 | Castano-Betancourt et al. [6] | | TLE3 | | 15q23 | | Hip JSW, mixed-gender | | < 5.0 × 10^−08^ | | 7.8 × 10^−06^ |
| rs12544183 | Castano-Betancourt et al. [6] | | RUNX1T1 | | 8q21 | | Hip JSW, mixed-gender | | < 5.0 × 10^−08^ | | 8.5 × 10^−06^ |
| rs7430431 | Zhao et al. [7] | | RTP3 | | 3p21 | | BR, mixed-gender | | < 4.2 × 10^−07^ | | 1.6 × 10^−07^ |
| rs16965654 | Hsuabd et al. [8] | | WSB1 | | 17q11 | | FNW, female | | < 4.3 × 10^−07^ | | < 4.2 × 10^−08^ |
|  |  |  |  |  |  |  | FNW, mixed-gender | | < 4.3 × 10^−07^ | | 6.9 × 10^−10^ |
| rs11573709 | Hsuabd et al. [8] | | RAD23 | | 9q31 | | FNSA, male | | < 4.3 × 10^−07^ | | < 2.4 × 10^−07^ |
| rs2278729 | Hsuabd et al. [8] | | TBC1D8 | | 2q11 | | FNSA, male | | < 4.3 × 10^−07^ | | 1.5 × 10^−07^ |
| rs7227401 | Hsuabd et al. [8] | | OSBPL1A | | 18q11 | | FNW, male | | < 4.3 × 10^−07^ | | 4.2 × 10^−07^ |
| rs494453 | Hsuabd et al. [8] | | RAP1A | | 1p13 | | FNW, female | | < 4.3 × 10^−07^ | | 2.8 × 10^−07^ |
|  |  |  |  |  |  |  | FNW, mixed-gender | | < 4.3 × 10^−07^ | | 3.6 × 10^−08^ |
| rs12885300 | Waarsing et al. [9] | | DIO2 | | 14q31 | | SSM hip joint, mixed-gender | | < (0.01-0.003)^1^ | | 0.005 |
| rs284857 | Hsu et al. [10] | | CYP17A1 | | 10q24 | | FNAL, mixed-gender | | < 5.0 × 10^−08^ | | 2.1 × 10^−08^ |
| rs10934815 | Hsu et al. [10] | | GPR175 | | 3q21 | | FNSA, mixed-gender | | < 5.0 × 10^−08^ | | 3.2 × 10^−07^ |
| rs845896 | Hsu et al. [10] | | FOXQ1 | | 6p25 | | FNSA, female | | < 5.0 × 10^−08^ | | 1.9 × 10^−07^ |
| rs1516893 | Hsu et al. [10] | | TMEM38B | | 9q31 | | FNAL, mixed-gender | | < 5.0 × 10^−08^ | | 4.0 × 10^−07^ |
| rs1953999 | Hsu et al. [10] | | OR4K14 | | 14q11 | | NNSM, female | | < 5.0 × 10^−08^ | | 6.8 × 10^−07^ |
| rs143383 | Southam et al. [11] | | GDF5 | | 20q11 | | Hip OA, mixed-gender | | < 0.05 | | 0.01 |
| rs11842874 | Day-Williams et al. [12] | | MCF2L | | 13q34 | | Hip OA, mixed-gender | | < 5.0 × 10^−08^ | | 2.1 × 10^−08^ |
| rs12901499 | Valdes et al. [13] | | SMAD3 | | 15q22 | | THR, mixed-gender | | < 0.05 | | 0.021 |
| rs4907986 | Rodriguez-Fontenla et al. [14] | | COL11A1 | | 1p21 | | Hip OA, mixed-gender | | < 1.58 × 10^−05^ | | 1.3 × 10^−05^ |
| rs1241164 | Rodriguez-Fontenla et al. [14] | | COL11A1 | | 1p21 | | Hip OA, mixed-gender | | < 1.58 × 10^−05^ | | 1.5 × 10^−05^ |
| rs4908291 | Rodriguez-Fontenla et al. [14] | | COL11A1 | | 1p21 | | Hip OA, female | | < 1.58 × 10^−05^ | | 1.3 × 10^−05^ |
| rs833058 | Rodriguez-Fontenla et al. [14] | | VEGF | | 6p21 | | Hip OA, male | | < 1.58 × 10^−05^ | | 1.4 × 10^−05^ |

^1^The authors did not strictly define a significance level but state that after adjustment for multiple testing it should be somewhere between 0.0125 and 0.0025.

Table S2: Candidate arcOGEN SNPs selected for this study: Original SNPs are those reported in the literature as associated with hip osteoarthritis or proximal femur morphology. “arcOGEN SNP in LD” lists the SNPs included in this study which includes those in high LD (defined as r^2^ or D’ > 0.85) with the “Original SNP”. “Gender-subset” specifies the gender strata where association was found in the original study and hence the gender stratum used in this study.

| Original SNP | arcOGEN SNP in LD | r^2^ | D’ | Gender-subset |  |
| --- | --- | --- | --- | --- | --- |
|  |  |  |  |  |  |
| rs6976 | rs6976 | - | - | mixed |  |
| rs4836732 | rs4836732 | - | - | female |  |
| rs9350591 | rs9350591 | - | - | mixed |  |
| rs10492367 | *not available* | - | - | - |  |
| rs835487 | rs835487 | - | - | mixed |  |
| rs12107036 | rs12107036 | - | - | female |  |
| rs8044769 | rs8044769 | - | - | female |  |
| rs10948172 | rs10948172 | - | - | male |  |
| rs12982744 | rs12974139 | 0.873 | 1.000 | male |  |
|  | rs2864419 | 0.867 | 0.964 |  |  |
| rs6094710 | *not available* | - | - | - |  |
| rs5009270 | rs5009270 | - | - | mixed |  |
| rs3757837 | rs7804804 | 0.398 | 1.000 | male |  |
| rs288326 | rs1561369 | 1.000 | 1.000 | female |  |
| rs7775 | rs16823787 | 1.000 | 1.000 | female |  |
|  | rs7593100 | 1.000 | 1.000 |  |  |
| rs1974201 | rs7754586 | 1.000 | 1.000 | mixed |  |
| rs4931462 | rs11051329 | 0.597 | 1.000 | mixed |  |
| rs10948155 | rs10948155 | - | - | mixed |  |
| rs11665774 | rs11666579 | 0.688 | 0.887 | mixed |  |
|  | rs8109373 | 0.250 | 1.000 |  |  |
| rs11738020 | *not available* | - | - | - |  |
| rs2380165 | rs8041787 | 0.886 | 0.960 | mixed |  |
| rs11206937 | rs2087796 | 1.000 | 1.000 | mixed |  |
| rs12907468 | rs2415060 | 0.648 | 0.868 | mixed |  |
| rs12544183 | *not available* | - | - | - |  |
| rs7430431 | rs7430431 | - | - | mixed |  |
| rs16965654 | *not available* | - | - | - |  |
| rs11573709 | rs11573709 | - | - | male |  |
| rs2278729 | rs6543018 | 0.737 | 1.000 | male |  |
| rs7227401 | rs1030110 | 0.927 | 0.963 | male |  |
| rs494453 | rs2477427 | 0.845 | 0.957 | mixed |  |
| rs12885300 | rs12885300 | - | - | mixed |  |
| rs284857 | rs619824 | 0.904 | 1.000 | mixed |  |
| rs10934815 | rs2044614 | 1.000 | 1.000 | mixed |  |
|  | rs4974421 | 1.000 | 1.000 |  |  |
| rs845896 | *not available* | - | - | - |  |
| rs1516893 | rs1516893 | - | - | mixed |  |
| rs1953999 | rs1953999 | - | - | female |  |
| rs143383 | rs6088813 | 0.929 | 1.000 | mixed |  |
| rs11842874 | rs11842874 | - | - | mixed |  |
| rs12901499 | rs12901499 | - | - | mixed |  |
| rs4907986 | rs11164665 | 0.869 | 0.964 | mixed |  |
| rs1241164 | rs1463039 | 1.000 | 1.000 | mixed |  |
|  | rs1241182 | 1.000 | 1.000 |  |  |
| rs4908291 | rs2376280 | 0.818 | 1.000 | female |  |
| rs833058 | rs699947 | 0.493 | 1.000 | male |  |

Table S3: Percentage of shape variation explained by the modes of the mixed-gender, female and male SSMs (n = number of subjects included in the model).


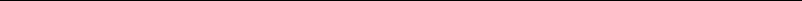


| SSM | Mixed-gender | Female | Male |
| --- | --- | --- | --- |
| mode | n=929 | n=570 | n=359 |
|  |  |  |  |
| 1 | 42.1% | 44.2% | 39.4% |
| 2 | 17.0% | 15.7% | 17.8% |
| 3 | 10.7% | 10.9% | 10.8% |
| 4 | 7.9% | 7.7% | 8.5% |
| 5 | 4.3% | 4.2% | 5.0% |
| 6 | 3.4% | 3.5% | 3.6% |
| 7 | 2.7% | 2.7% | 2.5% |
| 8 | 2.2% | 2.2% | 2.3% |
| 9 | 1.9% | 1.8% | 2.1% |
| 10 | 1.3% | 1.2% | 1.4% |
| 11 | 1.2% | 1.1% | 1.2% |
| 12 | 1.0% | 0.9% | 1.1% |
|  |  |  |  |

Table S4: Association between univariate SSM modes of the proximal femur and previously reported hip osteoarthritis or morphology susceptibility SNPs (MAF = minor allele frequency) after adjustment for height as well as for age, BMI and height. The expected level for significance was a familywise p-value < 0.05. All results were obtained running 100,000 permutation tests following an additive genetic model (if not stated otherwise).

| Chromo-some | SNP | Pointwise p-value | Familywise p-value | | Gender-subset | | SSM mode | | Minor allele | | MAF | | |  |
| --- | --- | --- | --- | --- | --- | --- | --- | --- | --- | --- | --- | --- | --- | --- |
|  | | |  | |  | |  | |  | |  | | |  |
| *After adjustment for height* | | | |  | |  | |  | |  | |  |  |  |
|  |  |  |  | |  | |  | |  | |  | | |  |
| 3 | rs6976 | 0.00159 | 0.03882 | | mixed-gender | | mode 7 | | T | | 0.40 | | |  |
| 3* | rs6976 | 0.00064 | 0.01417 | | mixed-gender | | mode 7 | | T | | 0.40 | | |  |
| 7 | rs5009270 | 0.00274 | 0.06168 | | mixed-gender | | mode 9 | | A | | 0.33 | | |  |
| 9 | rs4836732 | 0.00637 | 0.04495 | | females | | mode 5 | | T | | 0.48 | | |  |
|  | | |  | |  | |  | |  | |  | | |  |
| *After adjustment for age, BMI and height* | | | | | |  | |  | |  | |  |  |  |
|  |  |  |  | |  | |  | |  | |  | | |  |
| 3 | rs6976 | 0.00181 | 0.03704 | | mixed-gender | | mode 7 | | T | | 0.40 | | |  |
| 3* | rs6976 | 0.00061 | 0.01478 | | mixed-gender | | mode 7 | | T | | 0.40 | | |  |
| 7 | rs5009270 | 0.00714 | 0.1582 | | mixed-gender | | mode 9 | | A | | 0.33 | | |  |
| 9 | rs4836732 | 0.00476 | 0.03336 | | females | | mode 5 | | T | | 0.48 | | |  |

^*^ Results were obtained following a dominant rather than an additive genetic model.

Table S5: Multivariate association between the three most contributing SSM modes of the proximal femur and previously reported hip osteoarthritis or morphology susceptibility SNPs (MAF = minor allele frequency) after adjustment for height as well as for age, BMI and height. The expected level for significance after Bonferroni correction was a p-value < 0.001. All results were obtained following an additive genetic model.

| Chromo-some | SNP | p-value | Gender-subset | SSM modes | Minor allele | MAF |  |
| --- | --- | --- | --- | --- | --- | --- | --- |
|  | | |  |  |  |  |  |
| *After adjustment for height* | | |  |  |  |  |  |
|  |  |  |  |  |  |  |  |
| 3 | rs6976 | 0.00417 | mixed-gender | modes 5, 7, 10 | T | 0.40 |  |
| 7 | rs5009270 | 0.00066 | mixed-gender | modes 3, 4, 9 | A | 0.33 |  |
| 9 | rs4836732 | 0.00085 | females | modes 5, 8, 12 | T | 0.48 |  |
| 9 | rs1516893 | 0.0023 | mixed-gender | modes 7, 10, 11 | A | 0.12 |  |
| 15 | rs12901499 | 0.00206 | mixed-gender | modes 6, 7, 9 | A | 0.44 |  |
|  | | |  |  |  |  |  |
| *After adjustment for age, BMI and height* | | | |  |  |  |  |
|  |  |  |  |  |  |  |  |
| 3 | rs6976 | 0.00361 | mixed-gender | modes 5, 7, 10 | T | 0.40 |  |
| 7 | rs5009270 | 0.00040 | mixed-gender | modes 3, 4, 9 | A | 0.33 |  |
| 9 | rs4836732 | 0.00267 | females | modes 5, 8, 12 | T | 0.48 |  |
| 9 | rs1516893 | 0.00576 | mixed-gender | modes 7, 10, 11 | A | 0.12 |  |
| 15 | rs12901499 | 0.00210 | mixed-gender | modes 6, 7, 9 | A | 0.44 |  |


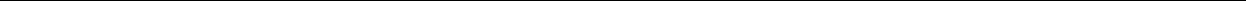


Table S6: Summary of power calculations for detecting an association with SSM modes 5 (effect size of rs4836732: -0.002899), 7 (effect size of rs6976: -0.001837) or 9 (effect size of rs5009270: -0.001611), assuming a Type I error rate of 5%. All effect sizes were taken from the univariate analyses following an additive genetic model (MAF = minor allele frequency).

| **Stratum** | **Sample size** | **SNP** | **MAF** | **Power to detect association** | | |
| --- | --- | --- | --- | --- | --- | --- |
|  |  |  |  | **Mode 5^a^** | **Mode 7^b^** | **Mode 9^c^** |
| mixed-gender | 929 | rs2087796 | 0.21 | 91.57% | 75.45% | 79.35% |
| mixed-gender | 929 | rs1241182 | 0.07 | 55.03% | 38.12% | 41.25% |
| mixed-gender | 929 | rs1463039 | 0.06 | 49.27% | 33.84% | 36.63% |
| mixed-gender | 929 | rs11164665 | 0.38 | 97.84% | 88.47% | 91.22% |
| mixed-gender | 929 | rs2477427 | 0.35 | 97.45% | 87.36% | 90.26% |
| mixed-gender | 929 | rs7430431 | 0.47 | 98.36% | 90.12% | 92.63% |
| mixed-gender | 929 | rs6976 | 0.40 | 98.02% | 89.03% | 91.70% |
| mixed-gender | 929 | rs2044614 | 0.09 | 64.83% | 45.99% | 49.62% |
| mixed-gender | 929 | rs4974421 | 0.09 | 64.83% | 45.99% | 49.62% |
| mixed-gender | 929 | rs10948155 | 0.38 | 97.84% | 88.47% | 91.22% |
| mixed-gender | 929 | rs9350591 | 0.13 | 78.59% | 58.93% | 63.04% |
| mixed-gender | 929 | rs7754586 | 0.18 | 88.22% | 70.46% | 74.55% |
| mixed-gender | 929 | rs5009270 | 0.33 | 97.10% | 86.42% | 89.44% |
| mixed-gender | 929 | rs1516893 | 0.12 | 75.78% | 56.03% | 60.08% |
| mixed-gender | 929 | rs619824 | 0.44 | 98.27% | 89.81% | 92.36% |
| mixed-gender | 929 | rs11051329 | 0.25 | 94.39% | 80.41% | 84.01% |
| mixed-gender | 929 | rs835487 | 0.38 | 97.84% | 88.47% | 91.22% |
| mixed-gender | 929 | rs11842874 | 0.07 | 55.03% | 38.12% | 41.25% |
| mixed-gender | 929 | rs12885300 | 0.38 | 97.84% | 88.47% | 91.22% |
| mixed-gender | 929 | rs12901499 | 0.44 | 98.27% | 89.81% | 92.36% |
| mixed-gender | 929 | rs2415060 | 0.32 | 96.89% | 85.89% | 88.96% |
| mixed-gender | 929 | rs8041787 | 0.28 | 95.73% | 83.17% | 86.53% |
| mixed-gender | 929 | rs8109373 | 0.20 | 90.60% | 73.92% | 77.90% |
| mixed-gender | 929 | rs11666579 | 0.47 | 98.36% | 90.12% | 92.63% |
| mixed-gender | 929 | rs6088813 | 0.36 | 97.60% | 87.77% | 90.61% |
| males | 359 | rs6543018 | 0.39 | 97.80% | 94.10% | 92.95% |
| males | 359 | rs699947 | 0.48 | 98.27% | 95.04% | 94.02% |
| males | 359 | rs10948172 | 0.36 | 97.45% | 93.40% | 92.18% |
| males | 359 | rs7804804 | 0.04 | 35.63% | 29.20% | 27.98% |
| males | 359 | rs11573709 | 0.23 | 92.86% | 85.92% | 84.11% |
| males | 359 | rs1030110 | 0.41 | 97.97% | 94.44% | 93.33% |
| males | 359 | rs2864419 | 0.42 | 98.04% | 94.58% | 93.49% |
| males | 359 | rs12974139 | 0.40 | 97.89% | 94.28% | 93.15% |
| females | 570 | rs2376280 | 0.39 | 97.94% | 88.77% | 92.47% |
| females | 570 | rs16823787 | 0.07 | 55.03% | 38.12% | 42.60% |
| females | 570 | rs7593100 | 0.07 | 55.03% | 38.12% | 42.60% |
| females | 570 | rs1561369 | 0.13 | 78.59% | 28.93% | 64.76% |
| females | 570 | rs12107036 | 0.45 | 98.31% | 89.94% | 93.40% |
| females | 570 | rs4836732 | 0.48 | 98.38% | 90.18% | 93.58% |
| females | 570 | rs1953999 | 0.45 | 98.31% | 89.94% | 93.40% |
| females | 570 | rs8044769 | 0.47 | 98.36% | 90.12% | 93.54% |

^a^ Based on a mean of 0.00 and an SD of 0.0153 (mixed-gender) / 0.0154 (males) / 0.0153 (females)

^b^ Based on a mean of 0.00 and an SD of 0.0122 (mixed-gender) / 0.0110 (males) / 0.0122 (females)

^c^ Based on a mean of 0.00 and an SD of 0.0102 (mixed-gender) / 0.0099 (males) / 0.0100 (females)

**Supplementary references**

[1] arcOGEN Consortium and arcOGEN Collaborators. Identification of new susceptibility loci for osteoarthritis (arcOGEN): a genome-wide association study. *Lancet* 2012;380 (9844):815–823.

[2] Evangelou E, Valdes AM, Castano-Betancourt MC, Doherty M, Doherty S, Esko T, et al. The DOT1L rs12982744 polymorphism is associated with osteoarthritis of the hip with genome-wide statistical significance in males. *Ann Rheum Dis* 2013;72(7):1264–1265.

[3] Evangelou E, [Kerkhof HJ](http://www.ncbi.nlm.nih.gov/pubmed?term=Kerkhof%20HJ%5BAuthor%5D&cauthor=true&cauthor_uid=23989986), [Styrkarsdottir U](http://www.ncbi.nlm.nih.gov/pubmed?term=Styrkarsdottir%20U%5BAuthor%5D&cauthor=true&cauthor_uid=23989986), [Ntzani EE](http://www.ncbi.nlm.nih.gov/pubmed?term=Ntzani%20EE%5BAuthor%5D&cauthor=true&cauthor_uid=23989986), [Bos SD](http://www.ncbi.nlm.nih.gov/pubmed?term=Bos%20SD%5BAuthor%5D&cauthor=true&cauthor_uid=23989986), [Esko T](http://www.ncbi.nlm.nih.gov/pubmed?term=Esko%20T%5BAuthor%5D&cauthor=true&cauthor_uid=23989986), et al. A meta-analysis of genome-wide association studies identifies novel variants associated with osteoarthritis of the hip. *Ann Rheum Dis* 2013;doi:10.1136/annrheumdis-2012-203114.

[4] Baker-Lepain JC, [Lynch JA](http://www.ncbi.nlm.nih.gov/pubmed?term=Lynch%20JA%5BAuthor%5D&cauthor=true&cauthor_uid=22544526), [Parimi N](http://www.ncbi.nlm.nih.gov/pubmed?term=Parimi%20N%5BAuthor%5D&cauthor=true&cauthor_uid=22544526), [McCulloch CE](http://www.ncbi.nlm.nih.gov/pubmed?term=McCulloch%20CE%5BAuthor%5D&cauthor=true&cauthor_uid=22544526), [Nevitt MC](http://www.ncbi.nlm.nih.gov/pubmed?term=Nevitt%20MC%5BAuthor%5D&cauthor=true&cauthor_uid=22544526), [Corr M](http://www.ncbi.nlm.nih.gov/pubmed?term=Corr%20M%5BAuthor%5D&cauthor=true&cauthor_uid=22544526), et al. Variant alleles of the Wnt antagonist FRZB are determinants of hip shape and modify the relationship between hip shape and osteoarthritis. *Arthritis Rheum* 2012;65(5):1457–1465.

[5] Cheung CL, [Livshits G](http://www.ncbi.nlm.nih.gov/pubmed?term=Livshits%20G%5BAuthor%5D&cauthor=true&cauthor_uid=19888898), [Zhou Y](http://www.ncbi.nlm.nih.gov/pubmed?term=Zhou%20Y%5BAuthor%5D&cauthor=true&cauthor_uid=19888898), [Meigs JB](http://www.ncbi.nlm.nih.gov/pubmed?term=Meigs%20JB%5BAuthor%5D&cauthor=true&cauthor_uid=19888898), [McAteer JB](http://www.ncbi.nlm.nih.gov/pubmed?term=McAteer%20JB%5BAuthor%5D&cauthor=true&cauthor_uid=19888898), [Florez JC](http://www.ncbi.nlm.nih.gov/pubmed?term=Florez%20JC%5BAuthor%5D&cauthor=true&cauthor_uid=19888898), et al. Hip geometry variation is associated with bone mineralization pathway gene variants: The Framingham Study. *J Bone Miner Res* 2010;25(7):1564–1571.

[6] Castano-Betancourt MC, [Cailotto F](http://www.ncbi.nlm.nih.gov/pubmed?term=Cailotto%20F%5BAuthor%5D&cauthor=true&cauthor_uid=22566624), [Kerkhof HJ](http://www.ncbi.nlm.nih.gov/pubmed?term=Kerkhof%20HJ%5BAuthor%5D&cauthor=true&cauthor_uid=22566624), [Cornelis FM](http://www.ncbi.nlm.nih.gov/pubmed?term=Cornelis%20FM%5BAuthor%5D&cauthor=true&cauthor_uid=22566624), [Doherty SA](http://www.ncbi.nlm.nih.gov/pubmed?term=Doherty%20SA%5BAuthor%5D&cauthor=true&cauthor_uid=22566624), [Hart DJ](http://www.ncbi.nlm.nih.gov/pubmed?term=Hart%20DJ%5BAuthor%5D&cauthor=true&cauthor_uid=22566624), et al. Genome-wide association and functional studies identify the DOT1L gene to be involved in cartilage thickness and hip osteoarthritis. *Proc Natl Acad Sci U S A* 2012;109(21):8218–23.

[7] Zhao LJ, [Liu XG](http://www.ncbi.nlm.nih.gov/pubmed?term=Liu%20XG%5BAuthor%5D&cauthor=true&cauthor_uid=20175129), [Liu YZ](http://www.ncbi.nlm.nih.gov/pubmed?term=Liu%20YZ%5BAuthor%5D&cauthor=true&cauthor_uid=20175129), [Liu YJ](http://www.ncbi.nlm.nih.gov/pubmed?term=Liu%20YJ%5BAuthor%5D&cauthor=true&cauthor_uid=20175129), [Papasian CJ](http://www.ncbi.nlm.nih.gov/pubmed?term=Papasian%20CJ%5BAuthor%5D&cauthor=true&cauthor_uid=20175129), [Sha BY](http://www.ncbi.nlm.nih.gov/pubmed?term=Sha%20BY%5BAuthor%5D&cauthor=true&cauthor_uid=20175129), et al. Genome-wide association study for femoral neck bone geometry. *J Bone Miner Res* 2010;25(2):320–329.

[8] Hsuabd YH, [Zillikens MC](http://www.ncbi.nlm.nih.gov/pubmed?term=Zillikens%20MC%5BAuthor%5D&cauthor=true&cauthor_uid=20548944), [Wilson SG](http://www.ncbi.nlm.nih.gov/pubmed?term=Wilson%20SG%5BAuthor%5D&cauthor=true&cauthor_uid=20548944), [Farber CR](http://www.ncbi.nlm.nih.gov/pubmed?term=Farber%20CR%5BAuthor%5D&cauthor=true&cauthor_uid=20548944), [Demissie S](http://www.ncbi.nlm.nih.gov/pubmed?term=Demissie%20S%5BAuthor%5D&cauthor=true&cauthor_uid=20548944), [Soranzo N](http://www.ncbi.nlm.nih.gov/pubmed?term=Soranzo%20N%5BAuthor%5D&cauthor=true&cauthor_uid=20548944), et al. An integration of genome-wide association study and gene expression profiling to prioritize the discovery of novel susceptibility loci for osteoporosis-related traits. *PLoS Genet* 2010;6(6):e1000977.

[9] Waarsing JH, [Kloppenburg M](http://www.ncbi.nlm.nih.gov/pubmed?term=Kloppenburg%20M%5BAuthor%5D&cauthor=true&cauthor_uid=21400473), [Slagboom PE](http://www.ncbi.nlm.nih.gov/pubmed?term=Slagboom%20PE%5BAuthor%5D&cauthor=true&cauthor_uid=21400473), [Kroon HM](http://www.ncbi.nlm.nih.gov/pubmed?term=Kroon%20HM%5BAuthor%5D&cauthor=true&cauthor_uid=21400473), [Houwing-Duistermaat JJ](http://www.ncbi.nlm.nih.gov/pubmed?term=Houwing-Duistermaat%20JJ%5BAuthor%5D&cauthor=true&cauthor_uid=21400473), [Weinans H](http://www.ncbi.nlm.nih.gov/pubmed?term=Weinans%20H%5BAuthor%5D&cauthor=true&cauthor_uid=21400473), et al. Osteoarthritis susceptibility genes influence the association between hip morphology and osteoarthritis. *Arthritis Rheum* 2011;63(5):1349–1354.

[10] Y. Hsu, T. Beck, S. Brown, et al. Meta-analysis of genome-wide association study (GWAS) identifies several genes for hip bone geometry in Caucasians: The Genetic Factors for Osteoporosis (GEFOS) Consortium. *J Bone Miner Res* 2010;25(Suppl 1):S19.

[11] Southam L, [Rodriguez-Lopez J](http://www.ncbi.nlm.nih.gov/pubmed?term=Rodriguez-Lopez%20J%5BAuthor%5D&cauthor=true&cauthor_uid=17616513), [Wilkins JM](http://www.ncbi.nlm.nih.gov/pubmed?term=Wilkins%20JM%5BAuthor%5D&cauthor=true&cauthor_uid=17616513), [Pombo-Suarez M](http://www.ncbi.nlm.nih.gov/pubmed?term=Pombo-Suarez%20M%5BAuthor%5D&cauthor=true&cauthor_uid=17616513), [Snelling S](http://www.ncbi.nlm.nih.gov/pubmed?term=Snelling%20S%5BAuthor%5D&cauthor=true&cauthor_uid=17616513), [Gomez-Reino JJ](http://www.ncbi.nlm.nih.gov/pubmed?term=Gomez-Reino%20JJ%5BAuthor%5D&cauthor=true&cauthor_uid=17616513), et al. An SNP in the 5’-UTR of GDF5 is associated with osteoarthritis susceptibility in Europeans and with in vivo differences in allelic expression in articular cartilage. *Hum Mol Genet* 2007;16(18):2226– 2232.

[12] Day-Williams AG, [Southam L](http://www.ncbi.nlm.nih.gov/pubmed?term=Southam%20L%5BAuthor%5D&cauthor=true&cauthor_uid=21871595), [Panoutsopoulou K](http://www.ncbi.nlm.nih.gov/pubmed?term=Panoutsopoulou%20K%5BAuthor%5D&cauthor=true&cauthor_uid=21871595), [Rayner NW](http://www.ncbi.nlm.nih.gov/pubmed?term=Rayner%20NW%5BAuthor%5D&cauthor=true&cauthor_uid=21871595), [Esko T](http://www.ncbi.nlm.nih.gov/pubmed?term=Esko%20T%5BAuthor%5D&cauthor=true&cauthor_uid=21871595), [Estrada K](http://www.ncbi.nlm.nih.gov/pubmed?term=Estrada%20K%5BAuthor%5D&cauthor=true&cauthor_uid=21871595), et al. A variant in MCF2L is associated with osteoarthritis. *Am J Hum Genet* 2011;89(3):446–450.

[13] Valdes AM, [Spector TD](http://www.ncbi.nlm.nih.gov/pubmed?term=Spector%20TD%5BAuthor%5D&cauthor=true&cauthor_uid=20506137), [Tamm A](http://www.ncbi.nlm.nih.gov/pubmed?term=Tamm%20A%5BAuthor%5D&cauthor=true&cauthor_uid=20506137), [Kisand K](http://www.ncbi.nlm.nih.gov/pubmed?term=Kisand%20K%5BAuthor%5D&cauthor=true&cauthor_uid=20506137), [Doherty SA](http://www.ncbi.nlm.nih.gov/pubmed?term=Doherty%20SA%5BAuthor%5D&cauthor=true&cauthor_uid=20506137), [Dennison EM](http://www.ncbi.nlm.nih.gov/pubmed?term=Dennison%20EM%5BAuthor%5D&cauthor=true&cauthor_uid=20506137), et al. Genetic variation in the SMAD3 gene is associated with hip and knee osteoarthritis. *Arthritis Rheum* 2010;62(8):2347–2352.

[14] Rodriguez-Fontenla C, [Calaza M](http://www.ncbi.nlm.nih.gov/pubmed?term=Calaza%20M%5BAuthor%5D&cauthor=true&cauthor_uid=24338622), [Evangelou E](http://www.ncbi.nlm.nih.gov/pubmed?term=Evangelou%20E%5BAuthor%5D&cauthor=true&cauthor_uid=24338622), [Valdes AM](http://www.ncbi.nlm.nih.gov/pubmed?term=Valdes%20AM%5BAuthor%5D&cauthor=true&cauthor_uid=24338622), [Arden N](http://www.ncbi.nlm.nih.gov/pubmed?term=Arden%20N%5BAuthor%5D&cauthor=true&cauthor_uid=24338622), [Blanco FJ](http://www.ncbi.nlm.nih.gov/pubmed?term=Blanco%20FJ%5BAuthor%5D&cauthor=true&cauthor_uid=24338622), et al. Assessment of Osteoarthritis Candidate Genes in a Meta-Analysis of Nine Genome-Wide Association Studies. *Arthritis Rheum* 2014;66(4):940–949.
